# The Sabbath

**Published:** 1859-09

**Authors:** 


### THE SABBATH.

The Sabbath is the poor man's day as much as the Bible is the poor man's book, one stands by and sustains the other, while both befriend the weary worker of the world. Not a man lives, of any observation, who is not conscious of a new alacrity for business on a Monday morning, when the Sabbath has been spent in rest, in quiet, and in temperance.

It is a very plausible argument in behalf of the poor, and the working people of cities, that once a week at least, they ought to have facilities by steamboats, and rail cars, and other forms of conveyance, by which they could get into the country and have a sight of the green trees, and breathe for a few moments the pure fresh air of heaven, and that it would be a humanity to open public parks to them near the large cities where they could peaceably walk in gardens, and wander among flowers, and gaze at paintings and statuary, and that cheap places of amusement and recreation should be opened for them, where they could enjoy themselves, and listen to elevating and refining music. These are the arguments used by a portion of the daily and weekly press of New York. It was in the advocacy of these things that a popular novelist made his great mistake, and ever since have calamities been coming on him, each or which has lowered his status, social and moral. The English government had the sagacity to see the falsity of such pleas, and resolutely refused to open the great public garden on the Sabbath, and license travelling to Sydenham pleasure grounds on that day.

Here in New York, where every body is a sovereign, and claims the right to do as he pleases, denouncing every thing as a puritanical prejudice which is contrary to his views, the experiment has been tried in various ways; "sacred" concerts were announced from time to time on the Sabbath day. But it was soon found that they were patronized only by rowdies and beer drinkers, and they fell through.

The other publicly advocated plan was, "let every rail-road and means of water communication to the country open frequent and cheap means of travel on Sundays. In this way a more healthy, moral and physical influence will be established, and our city purged of much of the riotous and ruinous debauch that now marks it on the Sabbath day. Let the good, the industrious, and the temperate be permitted to go forth, and hold converse with nature's charms." In less than a month the Sunday running boats were swarming from the city in every direction, when the same papers of the early Monday morning announced that "these ferry boats and Sunday excursions are denounced as great nuisances by every one who has had any experience in the police force—is the resort of desperate characters, who literally swarm the place every Sunday, drinking, fighting and yelling while there, and while in the boats, both going and returning, so that the sheriff has enrolled an especial police to guard the citizens from the depredations of these ruffians."

One is the theory fabricated in an editor's sanctum, the other is the comment made by that same editor when he became personally acquainted with the working of that theory. Thus it will always be that he who sets himself up to be wiser than the Bible, more liberal, more humane, more reasonable, will find himself mistaken. Be assured it is wisdom, it is health, it is thrift everywhere, to "remember the Sabbath day to keep it holy."

Meanwhile, let all the good be thankful for the announcement of the Monday morning papers of July 11th, 1859, that whereas the usual number of Sunday arrests before each of the four police courts for drunkenness has been from twenty to thirty, only about one dozen were taken up throughout the whole city, and nearly every dram shop was closed; and all this by the quiet, forbearing, and firm enforcement of existing laws on the part of the "Sabbath committee" composed of men, who for intelligence, business standing, social position and wealth, are among the very first. As long as special legislation, and party management, and epithet, and sarcasm, and bravado were the weapons of reformers, not a step forward was made; not only licensed liquor shops were opened on the Sabbath, but thousands boldly sold it on that day in defiance of law or license. But when a move was made for the suppression of the liquor traffic, and the better observance of the Sabbath day by gentlemen using gentlemanly means, respecting themselves and others likewise, courteously showing their rights, proving them, and in a firm and dignified manner insisting that those should be respected, see the change as to efficiency! and let it be a lesson for learning well, by all reformers of all times to come in proportion as they desire and hope for success.

